# Social determinants of self-rated health among Japanese mothers of children with disabilities

**DOI:** 10.1016/j.pmedr.2018.02.017

**Published:** 2018-03-08

**Authors:** Miyako Kimura

**Affiliations:** St. Marianna University, 2-16-1, Sugao, Miyamae-ku, Kawasaki-shi, Kanagawa, Japan

**Keywords:** Disabled children, Mothers, Social capital, Social determinants of health, Socioeconomic factors, Social isolation, Social support

## Abstract

Caregivers of children with disability are more likely to be affected by social determinants that lead to poor health. Additionally, a previous study revealed that although mothers of a single child with disability wanted to have another child, various obstacles including social, cultural, economic, and biological factors existed and some had to give up on having another child. Since the mental health and well-being of these mothers were poorer than those of mothers with multiple children with and without disabilities, such family composition may also affect maternal health. This study aimed to investigate and compare the social determinants of self-rated health of mothers only having children with disabilities and those having multiple children with and without disabilities. Through parents' associations of children with disabilities throughout Japan, 2311 self-administrated questionnaires were distributed to mothers of such children from January to March 2016. Out of the 1133 responses (return rate 49%), 1012 (43.8%) mothers of children with disabilities under 20 years of age were used for this study. Logistic regression showed that poor financial situation was most strongly related to poor self-rated health among all mothers. Other factors related to poor self-rated health were a lack of existence of child without disability, social isolation, low health consciousness, child's sex (girl), and severity of disability (mild/moderate). However, these relationships differ based on the existence of a child without disability. Investigating how socioeconomic and cultural conditions relate to family composition including child birth, and how they determine health is needed in the future.

## Highlights

• Social determinants of health of mothers of children with disabilities were found.
• Poor financial status was strongly associated with mothers' self-rated health.
• Social isolation and health consciousness had significant associations with health.
• Having multiple children with and without disability influenced mothers' health.
• Social capital was not related to mothers' self-rated health.

## 1 Introduction

The social determinants of health—that is, the conditions in which people are born, live, work, and age—are primarily responsible for health inequities according to the World Health Organization (WHO, n.d.). In other words, economically or socially disadvantaged people are more likely to suffer the burdens of social determinants that lead to poor health (WHO South Asia, 2009). This is clearly reflected in people with disability. Currently, >1 billion people (or about 15% of the world's population, i.e., one in seven people) have some form of disability, making it a global public health issue (WHO, 2015). Compared with their non-disabled peers, people with disabilities are more likely to experience poorer health outcomes as well as be affected by socioeconomic disadvantages (Emerson et al., 2009, Emerson et al., 2011).

The caregivers of such people with disabilities also experience burden. Caregivers of adults with disability tend to have worse employment opportunities and income, while caregivers of children with disability are more likely to experience divorce and delayed workforce entry (Emerson et al., 2009). Hock and Ahmedani (2012) also reported that the parents of children with autism spectrum disorder (ASD) were more likely to report poor neighborhood social capital, difficulty coping, lower levels of relationship satisfaction and mental health, and greater aggravation than did parents of children without ASD. Therefore, caring for people with disabilities might be associated with lower socioeconomic status and poorer health status.

Previously, my colleagues and I collected data from the mothers of children with intellectual disabilities (ID) in Japan, and found that although mothers' sense of coherence and subjective social capital predicted their mental health and positive changes (in their lives, health, and interpersonal relationships), financial difficulties were significantly and consistently related to poor maternal mental health and less positive change (Kimura and Yamazaki, 2016).

Moreover, although mothers of a single child with disability in the study wanted to have another child, various obstacles existed (e.g., recurrent risks, lack of support, and financial difficulty) and 42.5% had to give up on having another one (Kimura and Yamazaki, 2017). Since the mental health and well-being of these mothers were poorer than those of mothers with multiple children with and without disability, whether mothers could have another child without disability may be an important factor to determine mothers' health. However, whether these implications could be applied to other health measures, such as self-rated health, is unclear.

Self-rated health is considered an inclusive measure of health (Jylha, 2009), and a powerful predictor of future health and the utilization of health care services (Bath, 1999; Pappa and Niakas, 2006; Su et al., 2011). In addition, poor self-rated health is related to negative clinical outcomes (e.g., higher mortality and poorer QOL) and has been used as a screening tool for the assessment of general health (Jylha, 2009). Therefore, assessing self-rated health might be helpful for understanding the overall health of caregivers of children with disabilities.

This study aimed to investigate and compare the social determinants of self-rated health of mothers only having children with disabilities and those having multiple children with and without disabilities.

## 2 Method

### 2.1 Data source

The present study used secondary data, which were collected from January to March 2016 with the primary purpose of exploring the experiences related to pregnancy, child birth, and child-rearing among mothers of children with disabilities in Japan. I asked parents' associations in all 8 regions (47 prefectures) of Japan to cooperate with the survey, who then provided the number of possible participants in their corresponding region. In total, I distributed 2311 self-administrated questionnaires to the mothers of children with disabilities through these parents' associations, obtaining 1133 (49%) responses by postal mail. To be eligible for the study, participants had to be mothers of children with disabilities (intellectual disability, physical disability, chromosome abnormality, or internal impediment) and the child had to be <20 years of age. After excluding mothers of children with disabilities aged 20 years or over and other relatives, 1012 (43.8%) responses were considered in the analysis.

Social determinants of health included five determinant areas (economic stability, education, social and community context, health, neighborhood and environment) (Healthy People 2020, n.d.), but available four areas' data (excluded neighborhood and environment) were included in the analysis.

### 2.2 Measures

#### 2.2.1 Self-related health

Self-related health was assessed with a single question: “How do you evaluate your current health status?” Mothers responded using a five-point Likert scale (1 = “very good”, 2 = “fairly good”, 3 = “average”, 4 = “fairly bad”, and 5 = “bad”; Perlman and Bobak, 2008). Following past studies (Perlman and Bobak, 2008; Oshio and Kobayash, 2009), responses were dichotomized as “poor” (fairly bad or bad) and “not poor” (very good, fairly good, or average).

#### 2.2.2 Child's characteristics

The children's characteristics were assessed through child's age and sex, school level (under elementary, elementary/junior high school, high school or more), severity of disability, disability type, and child's behavioral difficulties. Severity of disability was assessed at the child consultation center of the municipality in which participants lived, and was divided into four categories—mild, moderate, severe, or profound. These were then dichotomized as “mild/moderate” and “severe/very severe.” Children's disability type was categorized as “ASD,” “Down syndrome” “other intellectual disabilities (ID) or chromosome abnormality,” and “internal impediment/physical disability.” Difficulty of child's behavior was evaluated with a single item: “Do you have extreme difficulty in dealing with your child's behavior?” This question was answered as “difficult to deal with” or “not difficult to deal with.”

#### 2.2.3 Sociodemographic variables including economic stability, education, and health

Mother's sociodemographic variables assessed included mother's age, employment status (“employed”: full time/part time/self-employed vs. “unemployed”: homemaker/others), marital status (“married” vs “currently not married”: unmarried/divorced/widowed), education level (“junior high school/high school,” “junior college/vocational school,” and “university/postgraduate”), family composition, perceived financial situation, and health consciousness. Family composition only focused on child's sibling composition, and dichotomized as “having child without disabilities” and “only having children with disabilities.” Perceived financial situation was evaluated with a 5-point Likert scale from 1 (poor) to 5 (rich); this was then dichotomized as “poor” (poor/fairly poor) and “not poor” (average/fairly rich/rich).

Health consciousness was assessed with a single item, where participants chose a response ranged from 1 (no longer pay attention to your health) to 5 (pay attention to your health). Their answers were then dichotomized as “not paying attention to own health” (no longer pay attention to your health/tend to not pay attention to your health) and “paying attention to own health” (yes and no/tend to pay attention to your health/pay attention to your health).

#### 2.2.4 Social and community context

Social and community context assessed included perceived social isolation, social support, and social capital.

Perceived social isolation was evaluated a single item: “I feel isolated from society.” Responses were made using a scale of 1 (agree) to 5 (disagree), and then dichotomized as “isolated” (agree/agree a little) and “not isolated” (neither agree nor disagree/disagree a little/disagree).

Social support was assessed as whether participants are able to obtain support from others (a spouse, other family members, peer group, specialists, teachers, or neighbors) or not. They responded to each question with “yes” or “no”.

Social capital was assessed in terms of subjective social capital, trust for neighbors, participation in community, and two single items related to social capital for child. The subjective social capital scale (Togari, 2006) was evaluated with 6 items assessing concepts like psychological sense of community (“Our neighbors are willing to help others who need support”) and neighborhood cohesion (“This neighborhood has a friendly atmosphere; we take care of others' homes when they are away”). Items are rated on a 5-point Likert scale from 1 (strongly disagree) to 5 (strongly agree), with higher scores indicating greater social capital. Mother's participation in community (“I am participating in activities held by the neighborhood community association, parent and teacher associations, or parents' associations of children with disabilities”) and trust for neighbors (“I think my neighbors are able to be trusted”), two single items related to social capital for child (“child with disability can participate in local events”; “child with disability regularly interact with children without disabilities”) were scored on a scale from 1 (strongly disagree) to 5 (strongly agree); the answers were then dichotomized as “yes” (strongly agree/agree/neither agree nor disagree) and “no” (strongly disagree/disagree).

#### 2.2.5 Statistical analysis

IBM SPSS Statistics 21 for Windows (IBM, Armonk, New York, USA) was used for all statistical analyses, with an alpha of 0.05 set as the level of significance. I examined differences in self-rated health (poor vs. not poor) and family composition (“having child without disability” vs. “only having children with disabilities”) according to each variable using the chi-square test and independent *t*-test. All missing data were treated as missing. To investigate the determinants of poor self-rated health (the dependent variable), logistic regression analyses (univariate and multivariate) were performed to calculate the odds ratios (ORs) and the adjusted odds ratios (AORs).

#### 2.2.6 Ethical considerations

This study was approved by the ethics committee at the Department of Medicine at my affiliated university. Returning the questionnaire indicated informed consent.

## 3 Results

### 3.1 Characteristics

Table 1 shows comparisons of participant characteristics by self-rated health. The distribution of answers for self-rated health was as follows: “very good” (n = 119, 11.8%), “fairly good” (n = 335, 33.1%), “average” (n = 418, 41.3%), “fairly bad” (n = 128, 12.6%), and “bad” (n = 12, 1.2%). When these answers were categorized, 140 mothers had poor self-rated health (13.8%) and 872 had not poor self-rated health (86.2%) (see the notes of Table 1). As for the comparison, mothers with the following characteristics tended to report poor self-rated health: having a girl (*p* < .05); having a child with higher educational level (i.e., high school or more) (*p* < .05); only having children with disabilities (*p* < .001); having two or more children with disabilities; having difficulty in dealing with the child's behavior (*p* < .05); currently being not married (*p* < .01) and being less educated (i.e., junior high/high school) (*p* < .05); had a poorer financial situation (*p* < .001); having low health consciousness (not paying attention to own health (*p* < .001); were socially isolated (*p* < .001); could not obtain support from their spouse (*p* < .01) or family members (*p* < .01). Furthermore, mothers with poor self-rated health were more likely to report lower subjective social capital (*p* < .01; Cronbach's alpha = 0.77, M = 20.2, SD = 4.3 in this study), as were mothers who did not trust their neighbors (*p* < .01).

**Table 1:** Characteristics of participants by self-rated health (n = 1021).

Variables	Self-related health			
	Not poor %		Poor %	
	N	(Mean)	N	(Mean)
Child's age (range 0–19)	872	(12.4)	140	(12.6)
Child's sex				
Girl	241	82.8	50	17.2⁎
Boy	631	87.5	90	12.5
Child's school level				
Under elementary school	66	88.0	9	12.0⁎
Elementary/junior high school	544	86.5	85	13.5
High school or more	262	85.1	46	14.9
Number of disabled child in family				
One	775	87.1	115	12.9⁎
Two or more	97	79.5	25	20.5
Severity of child's disability				
Mild/moderate	447	84.3	83	15.7
Severe/very severe	425	88.2	57	11.8
Child's disability type				
ASD	514	85.8	85	14.2
Down syndrome	133	89.9	15	10.1
Other ID or chromosome abnormality	192	86.5	30	13.5
Internal impediment/physical disability	16	72.7	6	27.3
Difficulty of child's behavior				
Difficult to deal with	370	83.3	74	16.7⁎
Not difficult to deal with	487	88.5	63	11.5
Mother's age (range 24–61)	860	(44.5)	137	(45.2)
Employment status				
Employed	412	88.2	55	11.8
Unemployed	455	84.4	84	15.6
Marital status				
Married	822	87.1	122	12.9⁎⁎
Currently not married	50	73.5	18	26.5
Education level				
Junior high/high school	252	81.6	57	18.4⁎
Junior college/vocational school	387	87.8	54	12.2
University/postgraduate	232	88.9	29	11.1
Family composition				
Having children WD	625	89.3	75	10.7⁎⁎⁎
Only having children with disabilities	242	79.1	64	20.9
Financial situation				
Not poor	699	90.5	73	9.5⁎⁎⁎
Poor	170	71.7	67	28.3
Health consciousness				
Paying attention to own health	831	87.3	121	12.7⁎⁎⁎
Not paying attention to own health	35	67.3	17	32.7
Perceived social isolation				
Not isolated	812	87.7	114	12.3⁎⁎⁎
Isolated	56	68.3	26	31.7
Obtain support from spouse				
Yes	718	87.9	99	12.1⁎⁎
No	154	79.0	41	21.0
Obtain support from family members				
Yes	738	87.6	104	12.4⁎⁎
No	134	78.8	36	21.2
Obtain support from peer group				
Yes	525	87.6	74	12.4
No	347	84.0	66	16.0
Obtain support from specialists				
Yes	711	86.0	116	14.0
No	161	87.0	24	13.0
Obtain support from teachers				
Yes	590	87.5	84	12.5
No	282	83.4	56	16.6
Obtain support from neighbors				
Yes	164	87.7	23	12.3
No	708	85.8	117	14.2
Subjective social capital (range7–30)	872	(20.4)	140	(19.3)⁎⁎
Trust for neighbors				
Yes	713	87.6	101	12.4⁎⁎
No	159	80.3	39	19.7
Own participation in community				
Yes	325	85.8	54	14.2
No	547	86.4	86	13.6
Child's participation in local events				
Yes	541	87.7	76	12.3
No	328	83.7	64	16.3
Child's interaction with children WD				
Yes	426	88.2	57	11.8
No	444	84.4	82	15.6

*t*-Test or chi-square test. ASD: autism spectrum disorders; ID: intellectual disabilities; WD: without disabilities.

Self-rated health were reported as “very good” (n = 119, 11.8%), “fairly good” (n = 335, 33.1%), “average” (n = 418, 41.3%), “fairly bad” (n = 128, 12.6%), and “bad” (n = 12, 1.2%), and these were summed as “poor” (n = 140, 13.8%) and “not poor” (n = 872, 86.2%).

All missing data were treated as missing.

⁎ *p* < .5.

⁎⁎ *p* < .01.

⁎⁎⁎ *p* < .001.

Table 2 shows the comparison of participant characteristics by family composition (the existence of a child without disability). Mothers only having children with disabilities were more likely to have a younger child with disability (*p* < .05), have two or more children with disability (*p* < .001), be unemployed (*p* < .001), have poor self-rated health (*p* < .05), not pay attention to own health (*p* < .05), and not obtain support from family members (*p* < .001).

**Table 2:** Characteristics of participants by family composition.

Variables	Having children WD (n = 700)		Only having children with disabilities (n = 306)	
	N	% (mean)	N	% (mean)
Child's age (range 0–19)	702	(12.6)	310	(12.0)⁎
Child's sex				
Girl	203	28.9	88	28.4
Boy	499	71.1	222	71.6
Child's school level				
Under elementary school	47	6.7	28	9.0
Elementary/junior high school	431	61.4	198	63.9
High school or more	224	31.9	84	27.1
Number of disabled child in family				
One	658	93.7	232	74.8⁎⁎⁎
Two or more	44	6.3	78	25.2
Severity of child's disability				
Mild/moderate	354	50.4	176	56.8
Severe/very severe	348	49.6	134	43.2
Child's disability type				
ASD	398	57.9	201	66.1
Down syndrome	107	15.6	41	13.5
Other ID or chromosome abnormality	168	24.5	54	17.8
Internal impediment/physical disability	14	2.0	8	2.6
Difficulty of child's behavior				
Difficult to deal with	393	57.2	157	51.1
Not difficult to deal with	294	42.8	150	48.9
Mother's age (range 24–61)	689	(44.4)	308	(45.1)
Employment status				
Employed	350	50.0	117	38.2⁎⁎⁎
Unemployed	350	50.0	189	61.8
Marital status				
Married	660	94.0	284	91.6
Currently not married	42	6.0	26	8.4
Education level				
Junior high/high school	216	30.8	93	30.0
Junior college/vocational school	312	44.5	129	41.6
University/postgraduate	173	24.7	88	28.4
Financial situation				
Not poor	543	77.6	229	74.1
Poor	157	22.4	80	25.9
Self-rated health				
Not poor	775	87.1	97	79.5⁎
Poor	115	12.9	25	20.5
Health consciousness				
Paying attention to own health	670	96.0	282	92.2⁎
Not paying attention to own health	28	4.0	24	7.8
Perceived social isolation				
Not isolated	646	92.3	280	90.9
Isolated	54	7.7	28	9.1
Obtain support from spouse				
Yes	575	81.9	242	78.1
No	127	18.1	68	21.9
Obtain support from family members				
Yes	621	88.5	221	71.3⁎⁎⁎
No	81	11.5	89	28.7
Obtain support from peer group				
Yes	409	58.3	190	61.3
No	293	41.7	120	38.7
Obtain support from specialists				
Yes	564	80.3	263	84.8
No	138	19.7	47	15.2
Obtain support from teachers				
Yes	466	66.4	208	67.1
No	236	33.6	102	32.9
Obtain support from neighbors				
Yes	136	19.4	51	16.5
No	566	80.6	259	83.5
Subjective social capital (range7–30)	695	(20.3)	307	(20.1)
Trust for neighbors				
Yes	576	82.1	238	76.8
No	126	17.9	72	23.2
Own participation in community				
Yes	261	37.2	118	38.1
No	441	62.8	192	61.9
Child's participation in local events				
Yes	437	62.4	180	58.3
No	263	37.6	129	41.7
Child's interaction with children WD				
Yes	341	48.6	142	46.3
No	361	51.4	165	53.7

*t*-Test or chi-square test. ASD: autism spectrum disorders; ID: intellectual disabilities; WD: without disabilities.

All missing data were treated as missing.

⁎ *p* < .5.

⁎⁎⁎ *p* < .001.

### 3.2 Factors related to poor self-rated health

The factors related to poor self-rated health via logistic regression analysis are shown in Table 3 (univariate analysis) and Table 4 (multivariate analysis). The age of children was correlated with their school level and mothers' age; it was removed from this analysis. In total (see Table 4), having a girl with disability (AOR = 1.62; 95% confidence interval CI: 1.04–2.54; *p* < .05), only having children with disabilities (AOR = 1.96; 95% CI: 1.24–3.10; *p* < .01), having a child with mild/moderate disability (AOR = 1.69; 95% CI: 1.07–2.67; *p* < .05), having a poor financial situation (AOR = 3.23; 95% CI: 2.05–5.11; *p* < .001), not paying attention to one's own health (AOR = 2.25; 95% CI: 1.07–4.72; *p* < .05), and perceived social isolation (AOR = 3.01; 95% CI: 1.65–5.51; *p* < .001) were significantly related to poor self-rated health.

**Table 3:** Factors related to self-related health (univariate analysis).

Variables		Total		Having children WD		Only having children with disabilities	
		OR	(95% CI)	OR	(95% CI)	OR	(95% CI)
Child's sex	Girl	1.46	(1.00–2.12)	1.71	(1.04–2.79)⁎	1.19	(0.65–2.16)
Child's school level (ref. under elementary school)	Elementary/junior high school	1.15	(0.55–2.39)	1.67	(0.50–5.59)	0.96	(0.36–2.52)
	High school or more	1.29	(0.60–2.76)	2.18	(0.64–7.48)	0.93	(0.33–2.65)
Number of disabled child in family	Two or more	1.74	(1.07–2.81)⁎	1.33	(0.54–3.25)	1.34	(0.73–2.47)
Severity of child's disability (ref. severe/very severe)	Mild/moderate	1.38	(0.96–1.99)	1.24	(0.77–2.01)	1.47	(0.83–2.59)
Child's disability type (ref. down syndrome)	ASD	1.47	(0.82–2.62)	1.11	(0.54–2.30)	2.02	(0.75–5.45)
	Other ID or chromosome abnormality	1.39	(0.72–2.68)	1.31	(0.59–2.92)	1.64	(0.51–5.22)
	Internal impediment/physical disability	3.32	(1.13–9.79)⁎	2.65	(0.63–11.09)	4.32	(0.78–23.88)
Difficulty of child's behavior	Difficult to deal with	1.55	(1.08–2.22)⁎	1.09	(0.67–1.76)	2.31	(1.30–4.11)⁎⁎
Mother's age (range 24–61)	Plus 1 year	1.02	(0.99–1.06)	1.04	(0.99–1.09)	1.00	(0.95–1.05)
Employment status	Unemployed	1.38	(0.96–1.99)	1.27	(0.79–2.05)	1.31	(0.73–2.34)
Marital status	Currently not married	2.43	(1.37–4.30)⁎⁎	4.82	(2.41–9.64)⁎⁎⁎	0.68	(0.23–2.05)
Education level (ref. university/postgraduate)	Junior high/high school	1.81	(1.12–2.93)⁎	1.52	(0.80–2.90)	2.49	(1.19–5.19)⁎
	Junior college/vocational school	1.12	(0.69–1.80)	1.08	(0.57–2.04)	1.25	(0.60–2.63)
Family composition	Only having children with disabilities	2.14	(1.49–3.08)⁎⁎⁎				
Financial situation	Poor	3.77	(2.60–5.47)⁎⁎⁎	3.51	(2.15–5.75)⁎⁎⁎	4.10	(2.29–7.35)⁎⁎⁎
Health consciousness	Not paying attention to own health	3.34	(1.81–6.14)⁎⁎⁎	1.00	(0.29–3.38)	6.66	(2.80–15.86)⁎⁎⁎
Perceived social isolation	Isolated	3.31	(2.00–5.48)⁎⁎⁎	4.11	(2.17–7.81)⁎⁎⁎	2.33	(1.02–5.32)⁎
Obtain support from spouse	Yes	1.93	(1.29–2.90)⁎⁎⁎	2.18	(1.28–3.71)⁎⁎	1.53	(0.82–2.87)
Obtain support from other family members	Yes	1.91	(1.25–2.91)⁎⁎	1.88	(1.00–3.55)⁎	1.40	(0.78–2.52)
Obtain support from peer group	Yes	1.35	(0.94–1.93)	1.15	(0.71–1.85)	1.80	(1.03–3.13)⁎
Obtain support from specialists	Yes	0.91	(0.57–1.46)	0.83	(0.44–1.55)	1.21	(0.58–2.54)
Obtain support from teachers	Yes	1.40	(0.97–2.01)	1.33	(0.82–2.17)	1.53	(0.87–2.70)
Obtain support from neighbors	Yes	1.18	(0.73–1.90)	0.97	(0.54–1.77)	1.48	(0.66–3.34)
Subjective social capital	Plus 1 point	0.95	(0.91–0.99)⁎⁎	0.96	(0.91–1.01)	0.94	(0.88–1.00)⁎
Trust for neighbors	Yes	1.73	(1.15–2.60)⁎⁎	1.37	(0.77–2.44)	2.05	(1.13–3.75)⁎
Own participation in community	Yes	0.96	(0.67–1.39)	1.05	(0.64–1.73)	0.87	(0.50–1.53)
Child's participation in local events	Yes	1.39	(0.97–1.99)	1.03	(0.63–1.68)	1.94	(1.11–3.38)⁎
Child's interaction with children WD	Yes	1.38	(0.96–1.99)	1.26	(0.78–2.40)	1.52	(0.87–2.68)

AOR: adjusted odds ratio; WD: without disabilities; ASD: Autism spectrum disorders; ID: intellectual disabilities.

All missing data were treated as missing.

⁎ *p* < .5.

⁎⁎ *p* < .01.

⁎⁎⁎ *p* < .001.

**Table 4:** Factors related to self-related health (multivariate analysis).

Variables		Total		Having children WD		Only having children with disabilities	
		AOR	(95% CI)	AOR	(95% CI)	AOR	(95% CI)
Child's sex	Girl	1.62	(1.04–2.54)*	1.95	(1.07–3.57)*	1.29	(0.61–2.76)
Child's school level (ref. under elementary school)	Elementary/junior high school	1.43	(0.55–3.70)	1.60	(0.31–8.15)	1.81	(0.43–7.58)
	High school or more	1.37	(0.47–4.00)	1.94	(0.34–11.23)	1.33	(0.24–7.26)
Number of disabled child in family	Two or more	0.82	(0.45–1.50)	0.69	(0.21–2.30)	0.72	(0.31–1.64)
Severity of child's disability (ref. severe/very severe)	Mild/moderate	1.69	(1.07–2.67)*	1.68	(0.91–3.09)	1.91	(0.86–4.24)
Child's disability type (ref. down syndrome)	ASD	1.07	(0.54–2.14)	1.02	(0.42–2.48)	1.17	(0.35–3.90)
	Other ID or chromosome abnormality	1.07	(0.50–2.28)	1.12	(0.43–2.92)	0.82	(0.20–3.31)
	Internal impediment/physical disability	2.82	(0.78–10.20)	3.26	(0.60–17.59)	3.77	(0.45–31.42)
Difficulty of child's behavior	Difficult to deal with	1.40	(0.89–2.19)	1.34	(0.73–2.44)	1.98	(0.93–4.22)
Mother's age (range 24–61)	Plus 1 year	1.03	(0.98–1.08)	1.03	(0.97–1.10)	1.02	(0.94–1.10)
Employment status	Unemployed	1.39	(0.89–2.18)	1.40	(0.77–2.53)	1.67	(0.77–3.64)
Marital status	Currently not married	1.96	(0.83–4.63)	5.75	(1.88–17.65)**	0.26	(0.05–1.42)
Education level (ref. university/postgraduate)	Junior high/high school	1.71	(0.95–3.07)	1.26	(0.56–2.84)	3.21	(1.24–8.30)*
	Junior college/vocational school	1.18	(0.67–2.07)	1.14	(0.54–2.44)	1.13	(0.44–2.89)
Family composition (ref. having children WD)	Only having children with disabilities	1.96	(1.24–3.10)**				
Financial situation	Poor	3.23	(2.05–5.11)***	2.95	(1.58–5.50)***	4.43	(2.03–9.68)***
Health consciousness	Not paying attention to own health	2.25	(1.07–4.72)*	0.81	(0.21–3.12)	4.37	(1.42–13.45)**
Perceived social isolation	Isolated	3.01	(1.65–5.51)***	4.44	(2.02–9.77)***	1.79	(0.59–5.38)
Obtain support from spouse	Yes	1.04	(0.56–1.93)	0.72	(3.00–1.75)	1.69	(0.64–4.45)
Obtain support from other family members	Yes	1.14	(0.67–1.94)	1.82	(0.82–4.06)	0.89	(0.40–1.96)
Obtain support from peer group	Yes	1.26	(0.78–2.02)	0.94	(0.48–1.84)	2.23	(1.03–4.82)*
Obtain support from specialists	Yes	0.55	(0.30–1.02)	0.58	(0.26–1.29)	0.38	(0.12–1.20)
Obtain support from teachers	Yes	1.37	(0.87–2.16)	1.45	(0.78–2.70)	1.40	(0.65–3.02)
Obtain support from neighbors	Yes	0.81	(0.45–1.47)	0.77	(0.37–1.61)	1.02	(0.32–3.26)
Subjective social capital	Plus 1 point	0.98	(0.93–1.04)	0.98	(0.91–1.06)	0.97	(0.88–1.08)
Trust for neighbors	Yes	1.01	(0.56–1.81)	1.02	(0.45–2.29)	0.68	(0.25–1.86)
Own participation in community	Yes	1.25	(0.77–2.04)	1.13	(0.57–2.23)	1.60	(0.72–3.58)
Child's participation in local events	Yes	1.08	(0.65–1.81)	0.78	(0.39–1.58)	1.74	(0.73–4.15)
Child's interaction with children WD	Yes	1.07	(0.66–1.74)	1.32	(0.70–2.51)	0.84	(0.35–2.02)

*p* < .1, *p < .5, **p < .01, ***p < .001; AOR: adjusted odds ratio; WD: without disabilities; ASD: Autism spectrum disorders; ID: intellectual disabilities.

All missing data were treated as missing.

Among mothers in the having child without disability group, having a girl (AOR = 1.95; 95% CI: 1.07–3.57; *p* < .05), being currently not married (AOR = 5.75; 95% CI: 1.88–17.65; *p* < .01), having a poor financial situation (AOR = 2.95; 95% CI: 1.58–5.50; *p* < .001), and perceived social isolation (AOR = 4.44; 95% CI: 2.02–9.77; *p* < .001) were related to poor self-rated health.

In contrast, among mothers in the only having children with disabilities group, being less educated (junior high/high school graduate) (AOR = 3.21; 95% CI:1.24–8.30; *p* < .05), having a poor financial situation (AOR = 4.43; 95% CI:2.03–9.68; *p* < .001) not paying attention to one's own health (AOR = 4.37; 95% confidence interval CI: 1.42–13.45; *p* < .01), and not obtaining support from peer group (AOR = 2.23; 95% CI: 1.03–4.82; *p* < .05) were related to poor self-rated health.

There is no significant relation between social capital (subjective social capital, trust for neighbors, own participation in community, and social capital for child) and self-rated health.

## 4 Discussion

This study investigated and compared the social determinants of self-rated health of mothers only having children with disabilities and those having multiple children with and without disabilities. Regardless of whether mothers had child without disabilities, poor financial situation was most strongly and consistently related to poor self-rated health. A previous study similarly revealed a strong relation between poorer psychological well-being (mental health and positive change) and poor financial situation (Kimura and Yamazaki, 2016). As such, I believe that financial situation is a powerful social determinant of both physical and mental health among the mothers of children with disabilities.

Family composition—the existence of a child without disability—could also be an important factor of mothers' self-rated health. Since the number of children with disabilities (i.e. two or more) was not significantly related to mothers' self-rated health in multivariate analysis, the existence of a child without disability may play a more powerful role in mothers' self-rated health. Compared to mothers who had multiple children with and without disabilities, those who only had children with disabilities were more likely to have a younger child with disability, have multiple children with disabilities, be unemployed, have poor self-rated health, not obtain support from family members, and not pay attention to own health. These findings were consistent with those of a previous study that found that mothers of single with ID had less hope and poorer mental health than those of children with and without ID (Kimura and Yamazaki, 2017). However, the study revealed that nearly half of the mothers of a single child with ID had decided to not have another child despite having the desire to do so. Thus, policy makers should consider ways to remove the obstacles to have another child and improve these mothers' health.

Through logistic regression analysis, remarkable differences were found in the comparison of the two groups of mothers only having children with disabilities and those with multiple children with and without disabilities. Socially isolated or currently not married mothers of multiple children with and without disability were more likely to report poor health and these relationships were not seen in mothers only having children with disabilities. In Japan, majority of children with disabilities are enrolled special-needs schools, which are separated from general public schools. Therefore, mothers who have children with and without disabilities may have more opportunities to interact with other mothers of only children without disabilities, and participate in various events in general public schools. If these mothers experience isolation resulting from lack of understanding of disability by other mothers, or have to raise multiple children with and without disabilities all by themselves because of being a single parent, it may affect their health.

On the other hand, mothers who only had children with disabilities may interact more with peer group, and if they could not obtain support from the group, it was related to poor self-rated health among these mothers. Another important difference was seen in health consciousness—specifically, not paying attention to one's own health— which was strongly associated with poor self-rated health among mothers only having children with disabilities. Since caring for children with disabilities require considerable time and effort, mothers could not have another child and might not have the time to be concerned about their own health.

Having a girl with a disability was also related to poor self-rated health among mothers of multiple children with and without disabilities. A possible reason is that girls with disabilities experience greater risk of facing sexual abuse than do boys with disabilities, and mothers might have more opportunities to accompany their daughters outside the house. In addition, mothers of multiple children with and without disabilities may have more opportunities to participate in events in general public schools especially those having girls with disabilities. Such situations might contribute for greater perceived exhaustion in mothers, and hence poorer self-rated health. However, more research examining how the sex of children with disabilities and their caregivers might be needed in Japan.

In total, having a child with mild/moderate disabilities showed a significant association with poor self-rated health. This may result from increased sample size when considering the total of mothers in the two groups. Potentially, this is because severity was evaluated by the child consultation center of each municipality in this study, which might not align with mothers' own perceptions. For example, if a child with a mild level shows hyperactivity, their mothers must often exert greater energy in caring for them (e.g., by chasing them around the house). In addition, some child with a mild/moderate level disability may have a sleep disorder, and these lead to mothers' sleep deprivation. Thus, levels of severity of disability may not always same as burden of caring child.

Another unexpected result was that social capital was not significantly associated with self-rated health in the multivariate analysis. This contradicts past studies on the general population in Japan, wherein a negative relation between poor self-rated health and social capital was reported (Hibino et al., 2012; Kobayashi et al., 2013). Moreover, when subjective social capital (using the same scale as in this study) was examined among mothers of children with ID, it was strongly and positively related to their positive change (Kimura and Yamazaki, 2016). Therefore, it is possible that social capital is related to only psychological well-being among mothers of children with disabilities. It is necessary to explore the mechanism of this result in detail in future studies.

This study revealed a relationship between self-rated health and social determinants among mothers of children with disabilities. However, there were also limitations. Since this study was cross-sectional, and I did not compare the results with mothers of children without disabilities, I cannot use this results to define the risk factors of self-rated health. In addition, the types of disabilities were not balanced. Moreover, income, which is likely a very important objective indicator, was not assessed in this study; I used only a single item (perceived financial situation) as a proxy. This is a crucial weakness of the current study. Furthermore, since this study used secondary data, various important social determinants such as smoking, drinking, physical activity and sociocultural status were not included, and there was a limited amount of regional information included in this study. Thus, collecting more information at both the individual and regional levels is needed in a future study.

## 5 Conclusion

This study showed that poor financial situation, perceived social isolation, lacking children without disability, low health consciousness (not paying attention to one's own health), child's sex (girl), and severity of disability (mild/moderate) were significantly related to poor self-rated health among Japanese mothers of children with disabilities. However, such relationships vary by the existence of a child without disability in addition to the child with disability. Thus, exploring how socioeconomic and cultural conditions relate to family composition including child birth, and how these determine health is needed in future study. In addition, to investigate the relationship between regional and country-level social determinants of health among caregivers of children with disabilities, a general population survey in Japan might be needed to include information about children with disabilities and their family compositions.

## Conflicts of interest

None.

## Fundings

This study was supported by JSPS KAKENHI Grant Number JP 26380716 and JP 17H02612.

## References

[bb0015] Bath P.A. (1999). Self-rated health as a risk factor for prescribed drug use and future health and social service use in older people. J. Gerontol. A Biol. Sci. Med. Sci..

[bb0020] Emerson E., Madden R., Robertson J., Graham H., Hatton C., Llewellyn G. (2009). Intellectual and Physical Disability, Social Mobility, Social Inclusion & Health. Centre for Disability Research, Lancaster University. http://eprints.lancs.ac.uk/26403/1/Disability_Social_Mobility_Social_Inclusion.pdf.

[bb0025] Emerson E., Madden R., Graham H., Llewellyn G., Hatton C., Robertson J. (2011). The health of disabled people and the social determinants of health. Public Health.

[bb0030] Healthy People 2020. n.d. Social Determinants of Health. Washington, DC: U.S. Department of Health and Human Services, Office of Disease Prevention and Health Promotion. http://www.healthypeople.gov/2020/topics-objectives/topic/social-determinants-health. (accessed 10 February 2018).

[bb0035] Hibino Y., Takaki J., Ogino K. (2012). The relationship between social capital and self-rated health in a Japanese population: a multilevel analysis. Environ. Health Prev. Med..

[bb0040] Hock R., Ahmedani B.K. (2012). Parent perceptions of autism severity: exploring the social ecological context. Disabil. Health J..

[bb0045] Jylha M. (2009). What is self-rated health and why does it predict mortality? Towards a unified conceptual model. Soc. Sci. Med..

[bb0005] Kimura M., Yamazaki Y. (2016). Mental health and positive change among Japanese mothers of children with intellectual disabilities: roles of sense of coherence and social capital. Res. Dev. Disabil..

[bb0010] Kimura M., Yamazaki Y. (2017). Having another child without intellectual disabilities: comparing mothers of a single child with disability and mothers of multiple children with and without disability. J. Intellect. Disabil..

[bb0050] Kobayashi T., Kawachi I., Iwase T., Suzuki E., Takao S. (2013). Individual-level social capital and self-rated health in Japan: an application of the Resource Generator. Soc. Sci. Med..

[bb0055] Oshio T., Kobayash M. (2009). Income inequality, area-level poverty, perceived aversion to inequality, and self-rated health in Japan. Soc. Sci. Med..

[bb0060] Pappa E., Niakas D. (2006). Assessment of health care needs and utilization in a mixed public-private system: the case of the Athens area. BMC Health Serv. Res..

[bb0065] Perlman F., Bobak M. (2008). Determinants of self-rated health and mortality in Russia – are they the same?. Int. J. Equity Health.

[bb0070] Su D., Richardson C., Wen M., Pagán J.A. (2011). Cross-border utilization of health care: evidence from a population-based study in South Texas. Health Serv. Res..

[bb0075] Togari T., Takegawa S., Hiraoka K., Ishida H., Yamazaki Y. (2006). An explanation of the development of subjective social capital indicator and relationship between self-rated health and subjective social capital. The Report of Grants-in-aid for Scientific Research (2002–2005 Fiscal Year). An Empirical Study of the Role of Socio Economic Factors in the Formation of Health Status.

[bb0080] World Health Organization (2015). WHO global disability action plan 2014–2021. Better health for all people with disability. http://apps.who.int/iris/bitstream/10665/199544/1/9789241509619_eng.pdf.

[bb0085] World Health Organization, n.d. Social determinants of health. http://www.who.int/social_determinants/sdh_definition/en/ (accessed 9 October 2017).

[bb0090] World Health Organization Regional office for South-East Asia (2009). Health inequities in the South-East Asia Region: selected country case studies. http://apps.who.int/iris/bitstream/10665/205241/1/B4288.pdf.

